# Supplementary material for: RNA sequence to structure analysis from comprehensive pairwise mutagenesis of multiple self-cleaving ribozymes
Source: eLife. 2023 Jan 19;12:e80360. doi: 10.7554/eLife.80360 (PMC9901934; doi:10.7554/eLife.80360)
Supplement: Supplementary file 1. [file elife-80360-supp1.docx]

| **Name** | **Sequence** | **Notes** |
| --- | --- | --- |
| **CPEB3 template** | GAACCGGACCGAAGCCCGATTTGGATCCGGCGAACCGGATCGA**ACAGCAGAATTCGCAGATTCACCAGAATCTGACAGGGGCTGCGACGTGAACGCTTCTGCTGTGGCCCCC**GAATGGTCCTTTTCCTATAGTGAGTCGTATTAGCCG | DNA template for in-vitro transcriptions. Bolded nucleotides indicate positions synthesized using doped phosphoramidites (3% mutation rate) |
| **HDV template** | GAACCGGACCGAAGCCCGATTTGGATCCGGCGAACCGGATCGA**TGGGTCCCATTCGCCATTACCGAGGGGACGGTCCCCTCGGAATGTTGCCCAGCCGGCGCCAGCGAGGAGGCTGGGACCATGCCGGCC**ATCAGGCCTATAGTGAGTCGTATTAGCCG | DNA template for in-vitro transcriptions. Bolded nucleotides indicate positions synthesized using doped phosphoramidites (3% mutation rate) |
| **Twister template** | GAACCGGACCGAAGCCCGATTTGGATCCGGCGAACCGGATCGA**CCGCCCCCTCCACTTTTATCCGGGCTTGGGACCGGCATTGGCAGTGTT**AGGCGGCCCTTTTCCTATAGTGAGTCGTATTAGCCG | DNA template for in-vitro transcriptions. Bolded nucleotides indicate positions synthesized using doped phosphoramidites (3% mutation rate) |
| **Hairpin template** | GAACCGGACCGAAGCCCGATTTGGATCCGGCGAACCGGATCGA**TACCAGGTAATATACCACAACGTGTGTTTCTCTGGTTCACTTCTCTCTTTCACGCGCACGTGAAAGAGGAC**TGTCATTTTCCTATAGTGAGTCGTATTAGCCG | DNA template for in-vitro transcriptions. Bolded nucleotides indicate positions synthesized using doped phosphoramidites (3% mutation rate) |
| **HH template** | GAACCGGACCGAAGCCCGATTTGGATCCGGCGAACCGGATCGA**CTGTTTCGTCCTCACGGACTCATCAGACCGGAAAGCACATCCGGT**GACAGTTTTCCTATAGTGAGTCGTATTAGCCG | DNA template for in-vitro transcriptions. Bolded nucleotides indicate positions synthesized using doped phosphoramidites (3% mutation rate) |
| **T7 top strand** | CGGCTAATACGACTCACTATAG | PCR primer |
| **RT primer** | GTCTCGTGGGCTCGGAGATGTGTATAAGAGACAGGAACCGGACCGAAGCCCG | PCR/RT primer |
| **TSO1** | TCGTCGGCAGCGTCAGATGTGTATAAGAGACAG GCATGCATGCATGCATGC rGrGrG | Phased template switching oligo 1 |
| **TSO2** | TCGTCGGCAGCGTCAGATGTGTATAAGAGACAG TGCATGCATGCATGC rGrGrG | Phased template switching oligo2 |
| **TSO3** | TCGTCGGCAGCGTCAGATGTGTATAAGAGACAG ATGCATGCATGC rGrGrG | Phased template switching oligo 3 |
| **TSO4** | TCGTCGGCAGCGTCAGATGTGTATAAGAGACAG CATGCATGC rGrGrG | Phased template switching oligo 4 |
